# *Bacillus cabrialesii*: Five Years of Research on a Novel Species of Biological Control and Plant Growth-Promoting Bacteria

**DOI:** 10.3390/plants12132419

**Published:** 2023-06-22

**Authors:** Karem Ma. Figueroa-Brambila, Alina Escalante-Beltrán, Amelia Cristina Montoya-Martínez, Alondra María Díaz-Rodríguez, Naomi Dayanna López-Montoya, Fannie Isela Parra-Cota, Sergio de los Santos-Villalobos

**Affiliations:** 1 Laboratorio de Biotecnología del Recurso Microbiano, Departamento de Ciencias Agronómicas y Veterinarias, Instituto Tecnológico de Sonora (ITSON), 5 de Febrero 818 Sur, Colonia Centro, Obregón 85000, Mexico; 2 Campo Experimental Norman E. Borlaug, Instituto Nacional De Investigaciones Forestales, Agrícolas y Pecuarias, Norman E. Borlaug s/n, Col. Centro, Obregón 85000, Mexico

**Keywords:** antimicrobial metabolites, *Bacillus cabrialesii*, bacterial inoculant, biological control, climate change, food security, plant growth-promoting bacteria, sustainable agriculture

## Abstract

*Bacillus cabrialesii* is a novel bacterial species isolated from wheat (*Triticum turgidum* L. subsp. *durum*) plants in the Yaqui Valley, Mexico, by our research team. Over years of research studying this strain at the cutting-edge level, it has shown different mechanisms of action. *B. cabrialesii* is strongly reported as a plant-growth-promoting bacterium and a biological control agent on wheat crops. Knowing this, *B. cabrialesii* has been brought from lab to field as part of a bacterial consortium, not to mention that there are ongoing investigations into formulating a cost-effective bioinoculant to increase the yield and/or quality of wheat. Moreover, studies of this novel species as a biocontrol agent in other crops (pepper, tomato, cucumber, and potato) are being carried out, with preliminary results that make *B. cabrialesii* a promising biological control agent, inhibiting the growth of phytopathogens. However, research into this bacterium has not only been reported in our country; there are many studies around the world in which promising native *Bacillus* strains end up being identified as *B. cabrialesii*, which reaffirms the fact that this bacterial species can promote plant growth and combat phytopathogens, showing great agrobiotechnological potential.

## 1. Introduction

The global population is currently increasing in impressive numbers, making food security a major challenge. The Food and Agriculture Organization (FAO) forecasts a population increase at an accelerated rate; it is expected to grow to almost 10 billion by 2050, which means that food demand will also continue increasing. It is estimated that by this year, the demand for cereals alone will reach 3 billion tons, and food production must increase by 70–100% [1].

In recent years, interest in the negative impacts of climate change, the reduced use of agrochemicals, and soil degradation have been increasing due to the environmental, economic, and health problems they entail, which, above all, threaten food production [2,3]. For example, climate change can interfere with plant physiology (transpiration, respiration, photosynthesis, and growth rates). Thus, the plants enter into a state of stress leading to a significant loss in crop yield; it is stated that biotic and abiotic stresses cause approximately 50–82% losses of crop productivity [4,5,6]. In addition, these consequences of climate change can lead to the emergence of strong, more infective pests and diseases, causing between 20 and 40% losses in agricultural production [2,4].

On the other hand, soil erosive processes intensify due to anthropogenic activities, leading to in situ consequences, including (1) the loss of the topsoil; (2) a lower water retention capacity; and (3) depletion of organic matter, nutrients, microbial resources, and fertility. Consequently, soil erosion emerges as the main cause of edaphic deterioration in the globe [7]. Currently, soil degradation affects 1.9 billion ha and is increasing rapidly at a rate of between 5 and 7 million ha per year, resulting in 80% of agricultural soils being moderately to severely degraded [8,9].

In this context, the development of innovative agricultural practices is needed to satisfy the sustainability of global food demand. Beneficial microorganisms, especially plant growth-promoting microorganisms (PGPM), play an essential role in maintaining functional and healthy agroecosystems [5,10]. These PGPM can enhance the acquisition of nutrients by plants, mitigate plant stress, and provide protection against pests and diseases through different mechanisms, improving crop yield and quality [4]. Therefore, the bioprospection of novel taxa of microorganisms with agrobiotechnological potential has been driven by the need to increase crop yields, while minimizing the use of agrochemicals and improving soil fertility [3,11].

**Figure 1:**
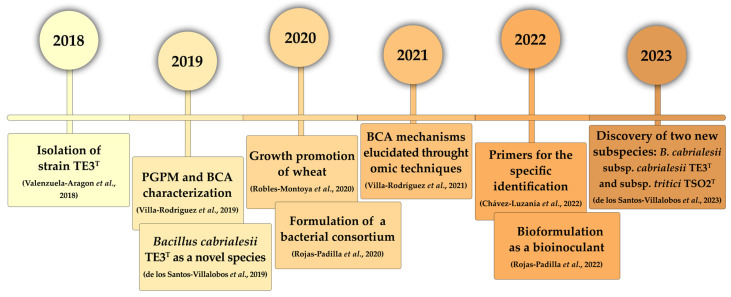
Timeline of advances in the study of strain *B. cabrialesii* TE3^T^ from its isolation to the discovery of two novel subspecies.

*Bacillus cabrialesii* TE3^T^ is an endophytic plant growth-promoting bacteria (PGPB) and biological control agent (BCA) with great agrobiotechnological potential which was isolated in 2018 from wheat plants in the Yaqui Valley, Mexico, by our research team [12,13]. Since that year, this novel bacterial taxon has been extensively studied due to several traits that make it well-suited for use in agriculture, including its mechanisms of action against phytopathogens and promotion of plant growth, as well as its potential to be used in the microbial inoculant bioformulation to be applied in several crops (Figure 1). Currently, this strain is cryopreserved in the Culture Collection of Native Endophytic and Soil Microorganisms (COLMENA, www.itson.mx/COLMENA, accessed on 21 June 2023), which is dedicated to the preservation of microorganisms as a soil conservation strategy, through the isolation, safeguarding, characterization, and typification of cultivable soil microbial resources [4].

This review presents the whole data generated from the isolation of *Bacillus cabrialesii* TE3^T^, summarizing all published findings and perspectives on this novel bacterial taxon worldwide. It presents relevant advances from its discovery and its positive impact on crop (mainly wheat) production in the Yaqui Valley, Mexico, the birthplace of the green revolution in the mid-twentieth century, for its migration to sustainable agriculture. Finally, this is a respectful acknowledgment and recognition to Dr. Juan José Peña Cabriales, a Mexican microbiologist who pioneered many investigations into plant-associated beneficial microorganisms and soil microbial ecology important to food supply and safety, as well as being an exemplary researcher, leader, and mentor of many researchers dedicated on studying the role of microorganisms in solving environmental issues worldwide.

## 2. Isolation and First Approaches as Plant Growth Promoting and Biological Control Bacterium

To mitigate the disturbances caused by conventional agricultural practices of wheat production in the Yaqui Valley, Mexico, and migrate to sustainable agriculture, the bioprospection of native beneficial microorganisms with high potential for microbial inoculant bioformulation begins in 2014 with our team (Microbial Research Biotechnology Laboratory -LBRM, www.itson.mx/LBRM, accessed on 21 June 2023). Strain TE3^T^ was first isolated in 2018 from wheat tissue as a result of a research project focused on developing an easy and feasible in vivo alternative to identifying promising PGPB by a technique called Plant-Assisted Selection (PAS) [14]. This was conducted by growing wheat under variable soil and climate conditions observed in the Yaqui Valley, using a growth chamber with the following parameters: 13 h of darkness at 14 °C, 2 h of light at 18 °C, 7 h of light at 25 °C, and 2 h of light at 18 °C.

**Figure 2:**
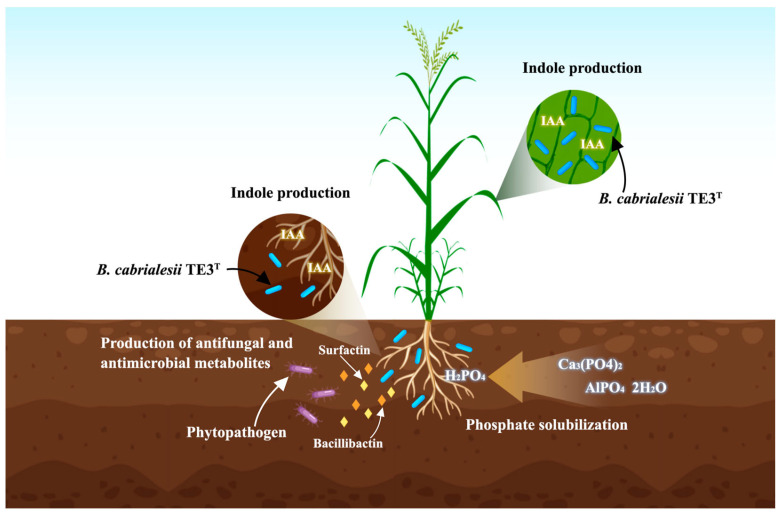
Summary of promising biological functions of *B. cabrialesii* TE3^T^ on the interaction with plants and phytopathogens.

Among the isolated bacteria, strain TE3^T^ showed the highest contents of chlorophyll when it was inoculated to wheat plants compared to the uninoculated control (57.2 SPAD Unit vs. 43.1 SPAD Unit). On the other hand, studying biochemical and stress tolerance traits, strain TE3^T^ was shown to have the ability to solubilize phosphate (43.2 ± 1.7%) and produce indoles (1.4 ± 0.1 ppm). In addition, this strain demonstrated high tolerance to thermal (43.5 °C), hydric (Polyethylene Glycol 6000 10%, −0.84 mPa), and saline (Sodium Chloride 5%, 6.8 dS m^−1^) conditions. Moreover, according to the molecular identification, based on sequencing the 16S rRNA gene, strain TE3^T^ was affiliated with the genus *Bacillus*. The results previously mentioned showed that strain TE3^T^ has promising metabolic and functional traits under several ecological scenarios during the plant-PGPB interaction (Figure 2) [14].

Later, Villa-Rodriguez et al. (2019) [12] focused on identifying and characterizing native wheat-associated bacteria (cryopreserved in COLMENA [4]) to find promising biological control strains that can be used as a sustainable alternative to control wheat spot blotch, caused by *Bipolaris sorokiniana* TPQ3 [15]. This phytopathogen generates economic losses for wheat production in warmer areas [16], initiating the disease with the adhesion of spores to the leaf, followed by germination and tissue penetration by appressorium [16,17]. Despite not inflicting significant losses to wheat yield in the Yaqui Valley, Mexico, the emergence of this phytopathogen is a serious biotic constraint due to climate change, highlighting the importance of establishing sustainable alternatives for disease management instead of chemical fungicides [15]. In this sense, the global biopesticide market has increased over the past few years, proving that the interest in beneficial plant-associated microorganisms and the study of their modes of biological control against phytopathogens has been enhanced [18].

Villa-Rodriguez et al. (2019) [12] evaluated the antifungal activity of strain TE3^T^ cell suspension (CS), cell-free culture filtrate (CF), its metabolic characterization, and in vivo biological control against spot blotch. In the identification of antagonistic bacteria, strain TE3^T^ proved to be a promising biocontrol strain of *B. sorokiniana* TPQ3. Moreover, its growth response under potentially stressful conditions observed in the Yaqui Valley, Mexico, was evaluated, being one of the strains that showed great capacity to grow under high temperatures (42 °C). Moreover, a hemolytic blood assay conducted on the strain did not reveal erythrocyte damage, indicating the absence of cytotoxic activity. The CF of strain TE3^T^ resulted in ∼98% of inhibition of *B. sorokiniana* TPQ3 under in vitro conditions, demonstrating that this strain produces extracellular antifungal metabolites that can effectively suppress the growth of this phytopathogen. In the in vivo biocontrol assay, CS and CF foliar application of strain TE3^T^ diminished the severity of spot blotch in wheat plants, which indicates that compounds contained in CF persist in leaf tissue, avoiding phytopathogen proliferation [12]. These results revealed the opportunity to explore the potential application of *B. cabrialesii* TE3^T^ as a biopesticide (Figure 2).

## 3. Taxonomic Affiliation of Strain TE3^T^ and Its Description as a Novel *Bacillus* Species

**Figure 3:**
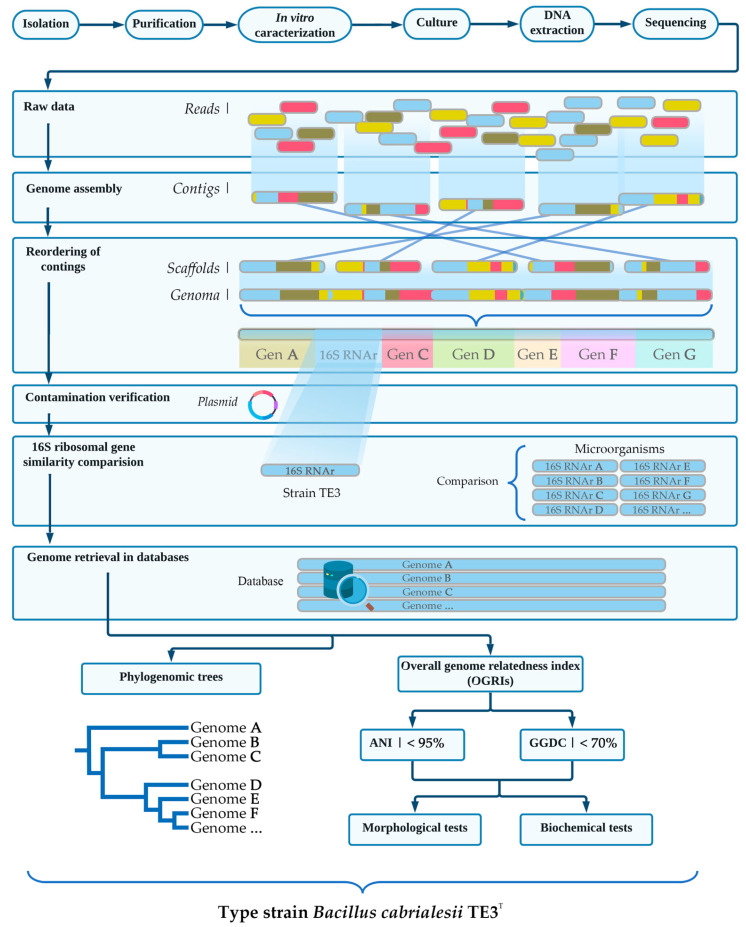
Taxonomical affiliation of *Bacillus cabrialesii* TE3^T^ using a polyphasic approach.

As mentioned before, Valenzuela-Aragón et al. (2018) [14] suggested that strain TE3^T^ belongs to the genus *Bacillus*, based on their sequencing of the full-length 16S rRNA gene, and months later, Villa-Rodriguez et al. (2019) [12] proposed its taxonomic affiliation to *Bacillus subtilis*. However, to define its taxonomical affiliation, a polyphasic characterization was used (Figure 3), for which the genome of strain TE3^T^ was sequenced using Illumina Sequencing by Synthesis (SBS) technology. Then, by using the EzTaxonserver and BLAST tools, the 16S rRNA gene sequence of strain TE3^T^ showed high similarity (100%) to *Bacillus subtilis* subsp. *Inaquosorum*. However, it is known that close relative strains of *Bacillus* are not reliably taxonomically distinguished only by their 16S rRNA gene sequences, but this analysis confirms its belonging to this genus [19,20]. Later, by using average nucleotide identity (ANI) analysis, strain TE3^T^ showed a maximum average value of 93.8% with *B. subtilis* subsp. *Inaquosorum*, followed by *B. subtilis* subsp. *Spizizenii* (93.6%), *B. tequilensis* (93.4%), and *B. subtilis* subsp. *subtilis* (92.3%), and using the Genome-to-Genome Distance Calculator (GGDC), the distances among genomes of strain TE3^T^ and closely related *Bacillus* species were below 70%. Based on these genomic analyses, strain TE3^T^ was distinguished as a novel species of the genus *Bacillus*, named *Bacillus cabrialesii* [21]. This novel species was named in honor of Dr. Juan Jose Peña Cabriales, a Mexican microbiologist who pioneered many investigations into plant-associated microorganisms and soil microbial ecology.

This discovery was supported by the fatty acid content analysis. The total cellular content of the fatty acids showed significant differences between strain TE3^T^ and its closest related species. These results reinforced the evidence to validate this novel *Bacillus* species. Finally, a macroscopic characterization of strain TE3^T^ was conducted after its incubation (24 h at 28 °C) on nutrient agar Petri dishes; white, circular, flat, motile colonies were observed, and TE3^T^ was classified as a Gram-stain-positive bacterium. In biochemical traits, strain TE3^T^ was sorted as catalase-positive, being able to hydrolyze starch and casein. It is also able to grow in the presence of lysozyme, produce acid from glucose, and produce indoles from tryptophan. Strain TE3^T^ is strictly aerobic; it reduces nitrate to nitrite, and citrate is utilized. In addition, its optimal temperature and pH were 28–30 °C and a range from 6.0 to 8.0, respectively. Furthermore, this strain was able to grow in the presence of 2% NaCl. In addition, strain TE3^T^ was microscopically analyzed using a scanning electron microscope, and it showed an average (*n* = 50) spore-forming rod-shaped cell length of 4.0 μm and cell width of 1.0 μm. The average spore length and width were 1.0 μm and 0.9 μm, respectively.

## 4. Bioprospecting *Bacillus cabrialesii* TE3^T^ as an Effective Bioinoculant for Wheat

### 4.1. Bacillus cabrialesii TE3^T^ Is a Plant Growth-Promoting Bacteria

In 2020, a native bacterial consortium of the genus *Bacillus*, isolated from the Yaqui Valley, Mexico, consisting of the strains *Priestia megaterium* TRQ8, *B. paralicheniformis* TRQ65, *B. cabrialesii* TE3^T^, and *Priestia megaterium* TSO9, was reported as a promoter of the growth of wheat seedlings [22]. There was recorded a significant increase in seven wheat biometric traits: length of the aerial part (28%), root length (25%), total length (28%), stem diameter (46%), circumference (50%), dry weight of the aerial part (72%), and biovolume index (57%), with respect to the control (uninoculated) treatment [22].

The authors suggested that the metabolic attributes shown by this consortium have a synergistic effect on the acquisition of nutrients and the regulation of phytohormones in inoculated plants. As a next step, it was proposed to evaluate the efficacy of this bacterial consortium under field conditions and explore its potential as a biofertilizer for its use in agriculture [22].

In the same year, Rojas-Padilla et al. [23] reported the growth promotion of wheat plants by co-inoculating native *Bacillus* strains isolated from the Yaqui Valley, Mexico. The evaluated bacterial strains were *B. cabrialesii* TE3^T^, *Priestia megaterium* TRQ8, and *B. paralicheniformis* TRQ65. In addition, these strains were metabolically characterized by functional activities associated with the promotion of plant growth (production of indoles, solubilization of insoluble phosphorus, and production of siderophores), where *B. cabrialesii* showed the capacity to produce indoles (8.21 ± 1.35 μg mL^−1^) and solubilize insoluble phosphorus (index = 1.43 ± 0.04) [23].

**Figure 4:**
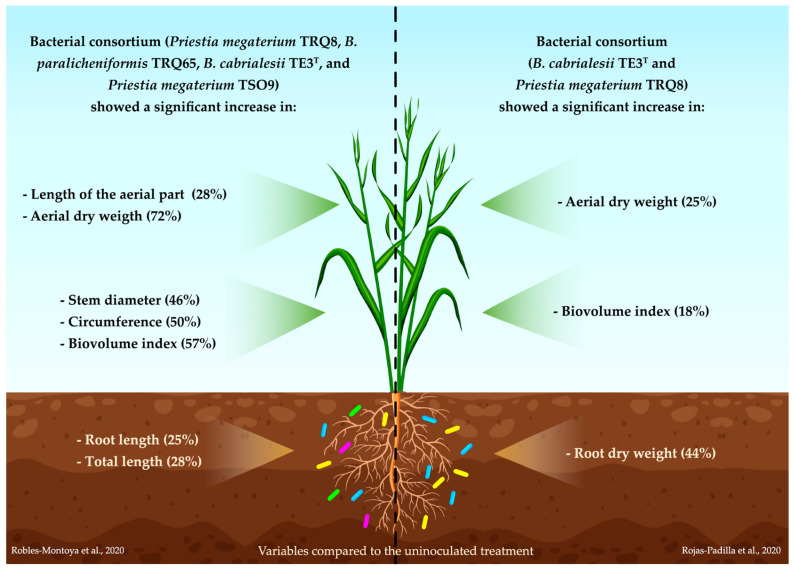
Increment of wheat biometric traits by the consortium composed of *Priestia megaterium* TRQ8, *B. paralicheniformis* TRQ65, *B. cabrialesii* TE3^T^, and *Priestia megaterium* TSO9 on the left-hand side; and increases in wheat biometric traits by the consortium of *Priestia megaterium* TRQ8 and *B. cabrialesii* TE3^T^ on the right-hand side [22,23].

The impact of the inoculation of these individual strains and in consortia was determined in wheat plants, simulating the edaphoclimatic conditions of the Yaqui Valley, Mexico, with a growth chamber (13 h of darkness at 14 °C, 2 h of light at 18 °C, 7 h of light at 25 °C, and 2 h of light at 18 °C). The measured morphometric variables were aerial and root length, aerial and root dry weight, and biovolume index (circumference x length). The TRQ8 + TE3^T^ consortium showed positive significant differences concerning the uninoculated treatment in aerial dry weight, root dry weight, and biovolume index (25%, 44%, and 18%, respectively), while the consortium of the three studied bacterial strains, TRQ8 + TRQ65 + TE3^T^, presented a tendency to increase the value of four variables (aerial length, aerial dry weight, root dry weight, and biovolume index); however, significant differences were not observed compared to the uninoculated plants [23]. These findings evidence the role of strain TE3^T^ as a promising active ingredient for the formulation of bacterial inoculants for increasing wheat production (Figure 4).

### 4.2. Deciphering Potential Mechanisms of Biological Control through Omic Techniques

**Figure 5:**
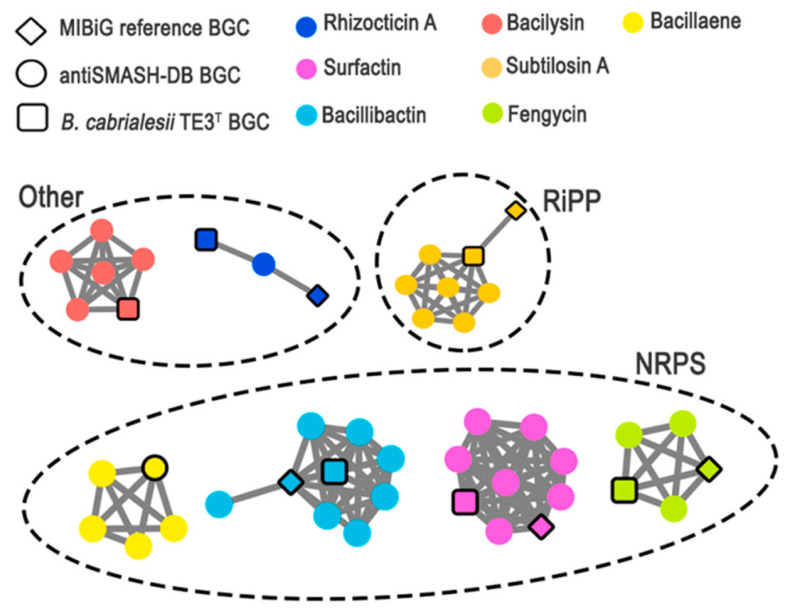
BiG-SCAPE network of BGCs identified in TE3^T^ genome containing validated BGC sequences from antiSMASH-DB and MIBiG databases. BGCs are clustered based on sequence similarity, where each node represents a BGC sequence. The node color represents the biosynthetic product, and its geometric form represents the origin of the BGC sequence (TE3^T^ genome, MIBiG, or antiSMASH-DB). Modified from Villa-Rodríguez et al. [13].

As important as metabolic characterization, genome mining can provide valuable insights into the capabilities of promising bacterial strains. Villa-Rodríguez et al. [13] reported that *Bacillus cabrialesii* TE3^T^ is a BCA and PGPB by showing the potential application of metabolites produced by this strain. In this study, *B. cabrialesii* TE3^T^ was the chosen strain for characterizing its cell-free culture filtrate (CF) and precipitated fraction (PF) by a metabolomic approach, due to its previously reported biological control activity against wheat spot blotch, caused by *Bipolaris sorokiniana* TPQ3 [12]. The results demonstrated that CF produced by *B. cabrialesii* TE3^T^ inhibits the growth of the studied phytopathogen. Moreover, by an integrated genomic–metabolomic approach, antifungal metabolites in CF and PF were explored. It was determined that strain TE3^T^ has the biosynthetic potential to produce a wide spectrum of antifungal (surfactin, fengycin, and rhizocticin A) and antibacterial metabolites (bacillaene, bacilysin, bacillibactin, and subtilosin A). The antifungal activity exhibited by *B. cabrialesii* TE3^T^ against *B. sorokiniana* TPQ3, which reduced spot blotch disease by 93%, was attributed to a lipopeptide complex of surfactin and fengycin homologs that disrupted the cytoplasmatic membrane (Table 1 and Figure 5) [13]. These results suggest the use of the strain TE3^T^ cells and/or its metabolites as potential biopesticides to control one of the most relevant phytopathogenic fungi, under current and future climatic scenarios, in the Yaqui Valley and regions with similar conditions.

### 4.3. An Efficient Tool for Detection and Monitoring Bacillus cabrialesii TE3^T^

**Figure 6:**
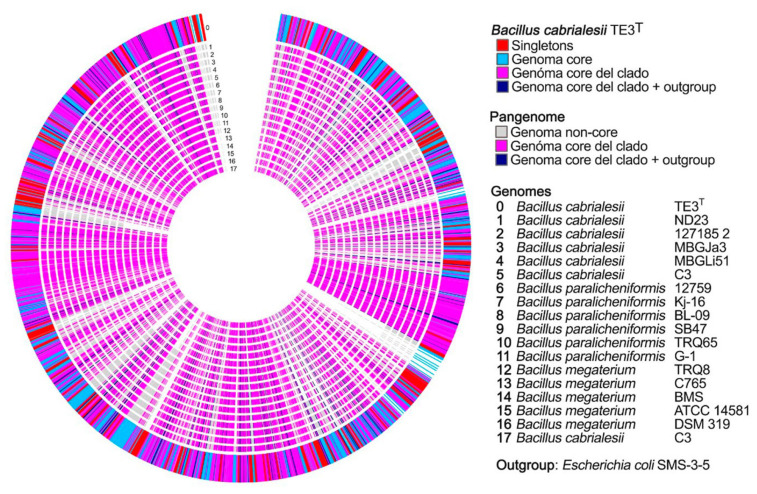
Pangenome constructed with strain TE3^T^ as the base, in which the following genomes were used: genome 0 as the studied strain; genomes 1 to 17 correspond to strains of *Bacillus cabrialesii*, *Priestia megaterium*, and *Bacillus paralicheniformis*, respectively; and a genome of *Escherichia coli* as outgroup. This visualization denotes the groups of the core genome, non-core genome, and singletons TE3^T^. Modified from Chávez-Luzania et al. [24].

**Figure 7:**
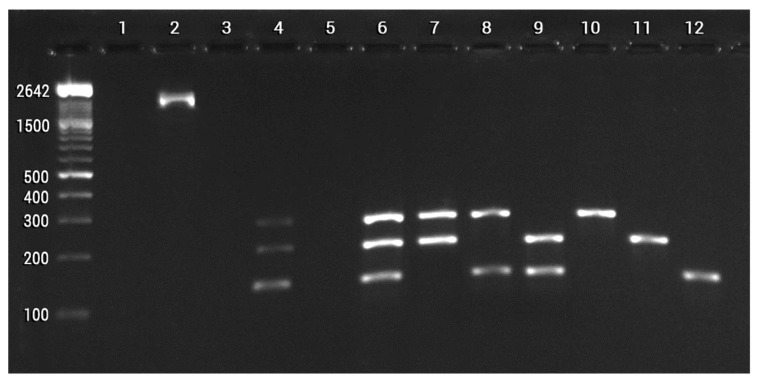
A total of 1.5% agarose gel electrophoresis using SB buffer and DNA Molecular Weight Marker XIV, showing amplifications of the 16S ribosomal gene using non-enriched soil DNA (lane 1: control; lane 2: DNA soil), and multiplex PCR amplifications with the primer sets TE3^T^_1218_F and TE3^T^_1218_R, TRQ8_1083_F, and TRQ8_1083_R, and TRQ65_6857_F and TRQ65_6857_R using different template DNA (lane 3: control; lane 4: enriched soil; lane 5: non-enriched soil; lane 6: TE3^T^, TRQ8 and TRQ65; lane 7: TE8 and TRQ65; lane 8: TE3^T^ and TRQ65; lane 9: TE3^T^ and TRQ8; lane 10: TRQ65; lane 11: TRQ8; and lane 12: TE3^T^). Modified from Chávez-Luzania et al. [24].

By 2022, Chávez-Luzanía et al. [24] reported the design of specific primers for the specific identification of strain TE3^T^, as well as other beneficial PGPB (*Priestia megaterium* TRQ8, and *Bacillus paralicheniformis* TRQ65), since they comprise a promising beneficial consortium for wheat. To this end, pangenomes were constructed to search for unique DNA sequences in each strain (Figure 6), and primer pairs were designed and synthesized. For the validation of the optimal primer pair set, high-quality DNA was extracted from pure bacterial and soil samples (Figure 7). Then, the designed primers were evaluated by uniplex and multiplex PCR. The results allowed the selection of primer pairs that specifically identified each strain alone or in a consortium. Thus, the use of pangenomes and discriminatory analysis, aided by specialized databases, offer tools for designing primers that allow for amplifying the target strains successfully. By using this tool, it will be possible to detect and trace the permanence of this strain when inoculated in the field under a single or consortium application [24].

### 4.4. Bioformulation of Bacillus cabrialesii TE3^T^ as a Bacterial Inoculant

**Figure 8:**
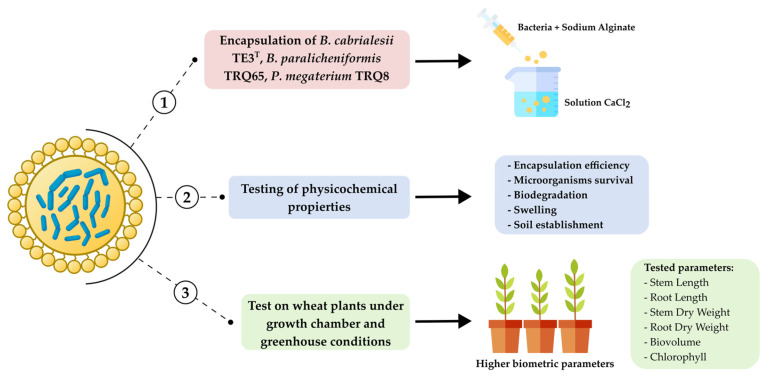
The strategy used for microencapsulation of strains and evaluation of their efficiency increasing biometric parameters on wheat plants.

The positive effect of inoculated PGPB on crop growth and yield remains an area of ongoing investigation, with several issues yet to be fully addressed. These include the viability of the inoculated bacterial strains and their ability to colonize the plant and/or soil. Therefore, in October 2022, a study was published on the microencapsulation of the strain *Bacillus cabrialesii* TE3^T^, *P. megaterium* TRQ8, and *B. paralicheniformis* TRQ65 using sodium alginate (Figure 8), achieving an encapsulation yield of 133.9% to 338.0% [25].

The encapsulation of bacteria in polymers is a technique that permits a controlled release of the microorganisms improving root colonization and providing physical protection of inoculated microorganisms against stressful environmental conditions [26]. The single and co-inoculation of alginate microbeads containing these promising strains exhibited a positive effect on wheat plants in growth chamber assays. For example, *B. cabrialesii* TE3^T^ showed a significant increase in root dry weight at 53.3%, while co-inoculation of encapsulated strains resulted in a significantly greater increase in stem and root length by 11.7% and 7.9%, respectively, compared to the uninoculated control treatment [25]. In addition, microencapsulation has been reported as an alternative to increasing the shelf life of microbial inoculants [27,28,29]. In previous trials, the concentration of strain TE3^T^ was maintained at 10^7^ CFU mg^−1^ for up to three months and reduced to 45% after one year of storage; however, in recording the stem and root length, stem and root dry weight, biovolume index, and chlorophyll variables by the application of strain TE3^T^ (44.7 ± 1.5 cm, 35.9 ± 1.5 cm, 1.0 ± 0.10 g, 0.42 ± 0.03 g, 8.1 ± 1.1, and 49.3 ± 0.5 SPAD Unit, respectively) and the consortium of strains TE3^T^ + TRQ8 (51.1 ± 0.9 cm, 37.1 ± 2.5 cm, 1.1 ± 0.05 g, 0.49 ± 0.03 g, 42.5 ± 1.2 and 50.5 ± 0.5 SPAD Unit, respectively), it was demonstrated that this bacterial population reduction in microbeads did not affect the plant growth-promoting activity as demonstrated in greenhouse assays [25].

### 4.5. Discovery of a Novel Subspecies of Bacillus cabrialesii

**Figure 9:**
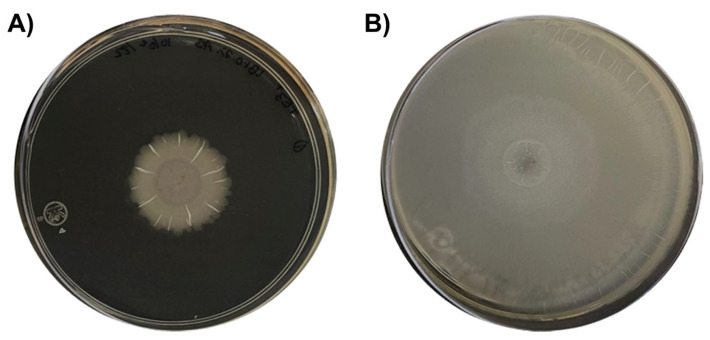
Growth differences between subspecies of *B. cabrialesii* cultured on Petri dishes containing Luria-Bertani broth supplemented with 0.7% bacteriologic agar: (**A**) *B. cabrialesii* subsp. *cabrialesii* TE3^T^ (swarming morphology presents radial strokes); (**B**) *B. cabrialesii* subsp. *tritici* TSO2^T^ (swarming morphology is shown as a featureless mat). Modified from de los Santos-Villalobos et al. [30].

Recently, our work has led us to discover a novel subspecies of *Bacillus cabrialesii*. The strain TSO2^T^ has been studied and tested as a PGPB and BCA, showing great capabilities, in some cases better than those of strain TE3^T^. Early taxonomic studies using the 16S rRNA gene had placed TSO2^T^ as *B. cabrialesii* but using a whole-genome approach with the overall genome-related indices—average nucleotide identity (ANI) and Genome-to-Genome Distance Calculator (GGDC)—it was revealed that the strain TSO2^T^ belonged to a novel subspecies different from strain TE3^T^. Therefore, two novel subspecies were reported: *B. cabrialesii* subsp. *cabrialesii* with strain TE3^T^, and *B. cabrialesii* subsp. *tritici* with strain TSO2^T^ (Figure 9) [30]. This discovery showed how much information and research is still required with respect to this bacterial species.

## 5. Current Research of *Bacillus cabrialesii* subsp. *cabrialesii* TE3^T^ Developed at the LBRM-COLMENA

### 5.1. Bacillus cabrialesii subsp. cabrialesii TE3^T^ as a Plant Growth Promotion

**Figure 10:**
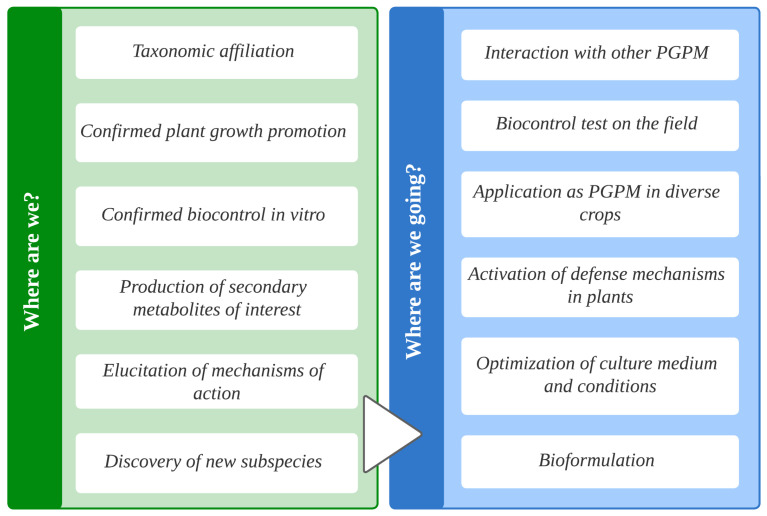
Progress to date and future studies to be carried out on *B. cabrialesii* subsp. *cabrialesii* TE3^T^.

The promising results observed in vitro and under greenhouse conditions, as reported by Robles-Montoya et al. [22] and Rojas-Padilla et al. [23] in 2020, have revealed the full potential of *B. cabrialesii* subsp. *cabrialesii* TE3^T^ as a bioinoculant for promoting plant growth. Therefore, efforts are currently underway to develop a cost-effective bioinoculant to increase the yield and quality of wheat crops. This involves optimizing culture media and growth conditions to obtain high biomass and spore yields; for example, the use of a minimal salt culture medium with 10 g/L of glucose or a minimal salt culture medium supplemented with monosodium glutamate, whichincreased the concentration of bio-active metabolites. In addition, the selection of the best carrier to increase the shelf life of this bioproduct is been studied (Figure 10).

In parallel, the effect of the application of *B. cabrialesii* subsp. *cabrialesii* TE3^T^ in consortium with other beneficial strains on wheat production, at the field level, is being studied to design alternatives that combine the use of these PGPB and lower doses of inorganic fertilizer. So far, the application of this consortium (including the strain TE3^T^, TRQ65, and TRQ8) in wheat, along with three different doses of nitrogen, has been evaluated. It has been found that the use of the studied bacterial consortium with a reduction of nitrogen fertilizer (urea) at doses of 0 or 120 kg N ha^−1^ increases the number of spikes/m^2^ by 25%, the crop yield by 15%, and improves grain quality by reducing the appearance of white belly and increasing the protein content [31].

On the other hand, the effective use of water and nitrogen greatly influences crop growth and yield and is critical for sustainable agricultural development [32]. Thus, the effect of the application of the *Bacillus*-based consortium (including the strain TE3^T^) on the efficient use of nitrogen and water in wheat is being evaluated. In this regard, to date, using a bacterial consortium composed of *B. cabrialesii* subsp. *cabrialesii* TE3^T^, *B. paralicheniformis* TRQ65, and *P. megaterium* TRQ8, an increase in nitrogen use efficiency in wheat of 10.8–14.4% has been found by reducing the conventional dose of nitrogen fertilization to 50% in combination with the inoculation of the bacterial consortium. This study was developed during the winter cycle, December–May 2018–2019, and the consortium was applied directly to the soil for TRQ65 and TRQ8, while TE3^T^ was foliar applied [33]. Similarly, in other agricultural cycles (December–May 2020–2021), higher wheat grain yields (~7.06 ton ha^−1^) and increases in water use efficiency of up to 24% have been obtained when conventional fertilization is combined with the soil application of the bacterial consortium composed by the same strains—TE3^T^, TRQ65, and TRQ8—while the control treatment (complete fertilization dose without the application of the bacterial consortium) resulted in a WUE (Water Use Efficiency) of 3.48 kg/m^3^ [34].

These results reveal that *B. cabrialesii* subsp. *cabrialesii* TE3^T^ can contribute to maintaining wheat crop productivity while minimizing overexploitation of water resources and N-driven environmental pollution.

### 5.2. Bacillus cabrialesii subsp. cabrialesii TE3^T^ as a Biological Control Agent

The potential of *B. cabrialesii* subsp. *cabrialesii* TE3^T^ as a biological control agent has been evaluated in vitro by Villa-Rodríguez et al. [12], and its mechanisms of action were elucidated in 2021 [13], where it was concluded that the biological control against *Bipolaris sorokiniana* TPQ3 was possible through the production of antifungal metabolites (surfactin and fengycin) in the cell-free culture filtrate (CF) and the precipitated fraction (PF). To further the knowledge of these biocontrol properties, new research is being conducted using diverse approaches and techniques.

To enhance the production of the antimicrobial metabolites produced by *B. cabrialesii*, different compositions of culture media are being evaluated to select one with higher (weight) precipitable metabolite production. Likewise, the biological control effect of the PF obtained from the selected culture media needs to be tested to ensure that the biocontrol properties are not lost. These experiments are being carried out in vitro with different culture media compositions, using *B. sorokiniana* as the model phytopathogen. The culture medium with a better result was a modified minimal salt medium containing monosodium glutamate. With this information, it would be possible to optimize the culture media for metabolite production of *B. cabrialesii* subsp. *cabrialesii* TE3^T^, enhancing the biological control properties of the bacteria [35].

Furthermore, the transcriptomic regulation of the gene clusters involved in the production of antimicrobial metabolites in *B. cabrialesii* subsp. *cabrialesii* TE3^T^ is being analyzed through mRNA-seq. Preliminary results showed greater differential gene expression during the stationary phase of growth of *B. cabrialesii* subsp. *cabrialesii* TE3^T^, which suggests a greater bioactivity associated with the biocontrol capacity seen in vitro against *B. sorokiniana*, for which the next step is the annotation of the transcripts to learn their biological functions [36].

On the other hand, it is known that PGPM and BCA can trigger the systemic resistance of plants, priming them before the arrival of pathogens [37,38]. The plant defense system related to the interaction with PGPM and BCA is complex and is mainly activated by microorganism-associated molecular patterns (MAMPs), which can be protein fragments essential for microorganisms, such as flagellins in bacteria or β-glucans in fungi, or metabolites produced by the microorganism [5]. In this sense, the induction of systemic resistance in wheat plants by the metabolites produced by *B. cabrialesii* subsp. *cabrialesii* TE3^T^ is being studied. For this, the cell-free culture filtrate of the growth phases (early exponential stage (EES), late exponential stage (LES), and stationary stage (SS)) of TE3^T^ was used to induce resistance in vitro against blotch spots in seedlings of wheat. Disinfected seedling roots were immersed in the studied three cell-free cultures, and the aerial part was immersed in a solution of *B*. *sorokiniana* spores. Preliminary results showed a decrease from 28.34 ± 6.79 to 3.97 ± 4.38 mm^2^ in the area of the lesion caused by spot blotch disease, representing a decrease from 22.15 to 2.98% of damaged plant areas when the roots were submerged in the ES-CF. These results are attributed to the induction of systemic resistance, due to the spatially separated inoculation of CF and pathogen [39]. Likewise, the transcriptional differential expression of defense-related genes will be analyzed to corroborate these results.

Having this information and knowledge, and to validate the use of *B. cabrialesii* TE3^T^ as BCA, field trials are being carried out. In a commercial wheat field, the application of *B. cabrialesii* subsp. *cabrialesii* TE3^T^ cellular biomass, CF, and whole culture (biomass and CF) are being tested as a biological control for fungal diseases such as spot blotch, rust, mildew, *Fusarium* head blight, root rot, or any fungal disease that may occur during the farming cycle. Likewise, plant growth-promoting and yield-increasing parameters are being monitored.

In addition, biocontrol tests are being conducted at the in vitro level to validate the use of *B. cabrialesii* subsp. *cabrialesii* TE3^T^ and other BCAs as biological control of vegetable diseases, such as *Fusarium* wilt in peppers and tomatoes, potato scab, and root-knot nematodes in tomatoes and cucumber. Preliminary results seem promising as *B. cabrialesii* subsp. *cabrialesii* TE3^T^ and other closely related strains have shown biocontrol activity against phytopathogens such as *Fusarium* spp., *Streptomyces* spp., and *Meloidogyne* spp.; however, the monitoring of these results will be carried out at the end of the agricultural cycle.

**Figure 11:**
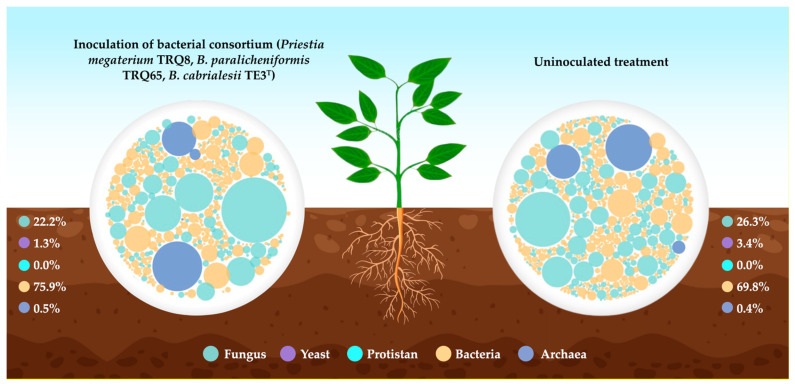
Microbial meta-taxonomic modulation in crop rhizosphere by the application of bacterial inoculants, including the novel subspecies *B. cabrialesii* subsp. *cabrialesii* TE3^T^.

Finally, a study that aims to identify the modulation of the edaphic microbiome in the Yaqui Valley, Mexico, by the application of three bioinoculants based on bacterial consortium in crops, is being carried out. The composition of the bacterial inoculants was based on eight beneficial native strains, including *B. cabrialesii* subsp. *cabrialesii* TE3^T^ and subsp. *tritici* TSO2^T^, *B. paralicheniformis* TRQ65, *Bacillus* sp. FSQ1 and TE5, *B. subtilis* TSO22, and *Priestia megaterium* TRQ8 and TSO9. Soil samples were collected from the rhizosphere of four commercial crops (jalapeno pepper, chickpea, corn, and summer corn) and two experimental crops of winter wheat and heat-stressed wheat (late planting), with and without the application of the bioinoculants, at different phenological stages. The soil samples were analyzed for identifying meta-taxonomic changes by sequencing the 16S rRNA genes for prokaryotes and ITS1 and ITS2 for eukaryotes using the Illumina Miseq platform. Results point to a marked modulation of fungal diversity in each of the studied crops and the phenological stage; otherwise, the bacterial diversity presents a homogeneous composition in all crops. Although robust statistical and ecological analyses are still underway, these results reinforce what has already been reported by other researchers and collaborators on the antifungal capacities of these bacterial strains (Figure 11) [40].

## 6. Research on *Bacillus cabrialesii* around the World

The study of *B. cabrialesii* is not limited to our country, since there are many studies from around the globe reporting the isolation and identification of strains belonging to this species from various crops and different locations, highlighting *B. cabrialesii* as a plant growth-promoting bacterium. Moreover, these studies also report this bacterial species as a biocontrol species against fungal and bacterial pathogens.

As shown in the previous paragraphs, there is a large line of research and investigation behind the discovery of *B. cabrialesii* subsp. *cabrialesii* TE3^T^. In the last three years, globally, several authors have reported the potential of *B. cabrialesii* strains as the best antagonists to combat various crop-related issues. In this instance, in January 2021, Zhou et al. [41] isolated 181 *Bacillus* strains from tomato rhizosphere soil and tomato plant tissue from the Netherlands and Spain and looked for biosynthetic gene clusters (BGCs) in these strains with the potential to inhibit plant-pathogens. After preliminary in vitro screening to identify potential Plant Growth Promoting Rhizobacteria (PGPR) against *Erwinia carotovora*, *Pseudomonas syringae*, *Rhizoctonia solani*, *Botrytis cinerea*, *Verticillium dahlia*, and *Phytophthora infestans*, they found four strains with major inhibition haloes on all pathogens. Further analysis revealed that strains BH5 and BH6 belonged to *B. cabrialesii*, based on ANI and digital DNA–DNA hybridization (dDDH) analysis [41].

In August of the same year, another work was published to study the mechanism between *B. cabrialesii* BH5 and pathogens in tomato plants and demonstrate the potential of this strain against phytopathogen by producing antifungal compounds [42]. The authors measured volatile organic compounds (VOC) produced by this strain on plant pathogens, and then antifungal compounds were identified. Finally, plants were grown in plastic pots for assessment of the strain BH5’s protective effect on tomato plants against *B. cinerea*. After evaluations, the main results showed that the antifungal activity of *B. cabrialesii* BH5 has a strong ability to inhibit the growth of *B. cinerea*, *F. culmorum*, *P. ultimum*, and *R. solani*, with mycelial inhibition rates of 61, 66, 50, and 63%, respectively. Volatile organic compounds produced in tests reached rates of 87, 32, 14, and 32% of inhibition toward the four pathogens. Referring to produced lipopeptides, a fengycin-like compound was found, but different from the discovered fengycin compounds reported until now. This compound was named fengycin H by the authors. It was found that fengycin H triggered the hyphal cell membrane defects and, consequently, cell death. Because of this, they suggest that this compound may be involved in the mechanism of elicitation of *B. cabrialesii* against *B. cinerea* [42].

In 2021, Kannan et al. [43] isolated six native *Trichoderma* and *Bacillus* strains from rice crops in India to prove their potential for growth promotion and to suppress *Xanthomonas oryzae pv. oryzae (Xoo)* and *R. solani*. The results confirmed that two native isolates, TAIK 1 and BIK 3, had the highest efficiency against the two pathogens. Strain similarity analysis revealed that BIK 3 had 97–98% similarity with *B. subtilis* IIRRCKB3. However, in 2022, the authors sequenced the whole genome of the studied isolates and identified the strain BIK 3 as a member of the species *B. cabrialesii* [44].

Other biocontrol studies have been carried out on wheat, Mulk et al. [45] evaluated the antagonistic activity of bacteria against wheat root rot fungi such as *F. oxysporum*, *F. moniliforme*, *M. phaseolina*, and *R. solani*. The most relevant results of this study demonstrated that two varieties of wheat (Sahar and Galaxy), treated with a consortium of *B. velezensis* SM-39 and *B. cabrialesii* SM-93, reduced the severity of *Fusarium* root rot in a range of 52–56% compared to uninoculated controls. These results were followed by strain *B. velezensis* SM-39 alone, which caused a decrease in the disease severity of 49%, and strain *B. cabrialesii* SM-93, which decreased the severity of *Fusarium* root rot in a range of 42–49% when compared to uninoculated controls. In a net house experiment, it was shown that *Bacillus velezensis* SM-39 and *Bacillus cabrialesii* SM-93 had a significant role in the suppression of *Fusarium* root rot severity in wheat, with percentages between 42 and 62% [45].

In the same year, Naseer et al. [46] isolated and identified vermi-bacteria associated with *Eisenia fetida* involved in heavy metal remediation and plant growth promotion. They reported that the strain MZ342760.1 belonged to *Bacillus cabrialesii*, comparing part of the nucleotide sequences by BLAST analysis at the National Center for Biotechnology Information (NCBI), finding 99.29% similarity and confirming through phylogenetic analysis constructed using the Maximum Likelihood method and Tamura-Nei model. This strain demonstrated via Atomic Absorption Spectroscopy that it was efficient in the removal of cadmium, chromium, and lead, with an accumulation of 4.50 ± 0.01 ppm, 4.24 ± 0.01 ppm, and 1.24 ± 0.01 ppm, respectively, indicating its potential to enhance plant growth and improve soil health by reducing heavy metal levels [44].

In a study by Rajer et al. [47], the ability of strains isolated from the rice rhizosphere to promote rice seedlings’ growth and suppress bacterial blight (BB) disease caused by *X. oryzae* pv. *oryzae* (*Xoo*) was evaluated. For this, the antagonistic activity of *Bacillus* strains was evaluated in vitro and under greenhouse conditions. The results showed that strain FA26 significantly enhanced rice seedlings by 108.70%, while also inhibiting all phytopathogens tested (*Sclerotinia sclerotiorum*, *R. solani*, *F. oxysporum*, *Phytophthora capsici*, *Magnaporthe oryzae*, *E. amylovora*, *Clavibacter michiganensis* spp. *sepedonicus*, and *Xoo*). Further analysis based on 16S rRNA and gyrB gene sequences revealed that strain FA26 had 100% and 97.05% similarity, respectively, to *Bacillus cabrialesii* subsp. *cabrialesii* TE3^T^. Based on these findings, the authors propose that strain FA26 is an effective biocontrol agent against BB under greenhouse conditions [47].

In 2022, Dabiré et al. [48] conducted a study to investigate the probiotic attributes of presumed-safe *Bacillus* spp. strains isolated from soumbala, a traditional fermented food from Burkina Faso. The study evaluated these strains for in vitro probiotic criteria, including tolerance to low pH, gastric juice, bile salts, phenol, intestinal juice, auto-aggregation capacity, cell surface hydrophobicity, antibiotic susceptibility, antimicrobial activity against foodborne pathogens, and biofilm production. At the same time, other properties were also assessed, including lipase, protease, amylase, and tannase activity, as well as poly-γ-glutamic acid (PGA) production and thermo-tolerance. From the six *Bacillus* strains isolated, F26 was identified as *B. cabrialesii*, with 100% similarity, using the EzBioCloud tool. Results showed that strain F26 stood out in all tolerance tests, as well as being one of the best in biofilm formation. In regard to PGA production, *B. cabrialesii* was also in the top three producer strains. In summary, it is suggested that *B. cabrialesii* F26 could serve as a potential source of probiotic starters, with commercial value for the production of high-quality soumbala because of its probiotic and technological characteristics [48].

Finally, at the end of 2022, Pawaskar et al. [49] evaluated the potential of halotolerant salt-pan bacteria as plant growth promoters and inhibitors of fungal pathogens affecting chili (*Capsicum annuum* L.) crops in saline soils. The method included sample collection and isolation of bacteria from salt pans and in vitro antifungal evaluation. Results demonstrated that strain MPSK109 showed the highest inhibition against *R. solani* (65.9%), *P. aphanidermatum* (56%), *F. oxysporum* (60.5%), and *F. pallidoroseum* (55%). This strain showed 100% similarity with *B. cabrialesii* based on partial 16S rRNA gene sequencing reported in the NCBI GenBank. These findings suggest that *B. cabrialesii* and other halotolerant isolates could grow under a wide range of pH, temperature, and NaCl concentrations, and exhibit plant growth-promoting attributes [49].

**Figure 12:**
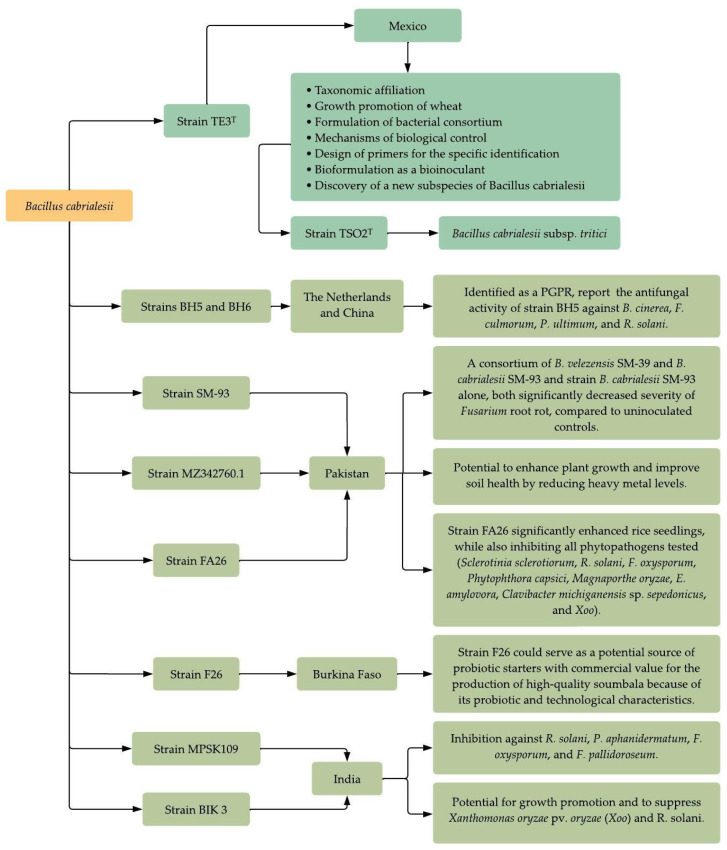
An insight into the research carried out with *Bacillus cabrialesii* around the globe.

Thus, from the discovery of *B. cabrialesii* subsp. *cabrialesii* TE3^T^, various studies conducted around the globe have shown the high potential of this novel *Bacillus* species to inhibit or suppress the presence of numerous pathogens in several crops. This species has been proposed for further experimentation to explore its application as a plant growth-promoting bacterium and biocontrol agent against phytopathogens. It has also been demonstrated to be effective in the bioremediation of heavy metals. All these discoveries present an opportunity to deepen and exploit the potential of *B. cabrialesii* as a beneficial bacterium in a wide range of agrobiotechnological applications (Figure 12).

## 7. Conclusions and Perspectives

Through the last five years of research, the agrobiotechnological potential of *B. cabrialesii* has been elucidated; it represents a powerful strategy for sustainable agriculture due to its capabilities as a plant growth promoter and biological control agent.

An interesting approach to studying *B. cabrialesii* involves utilizing transcriptomic and metabolomic techniques to elucidate the mechanisms involved in its interaction with other bacteria in a consortium. These omic techniques can also be employed to decipher its role in inducing systemic resistance in plants and its interaction with phytopathogenic fungi. Additionally, ongoing work is focused on the development of a cost-effective bioinoculant using *B. cabrialesii* to enhance the yield and quality of wheat crops. These efforts involve optimizing the culture media and growth conditions to achieve high biomass and spore yields, as well as increasing the concentration of bioactive metabolites. Furthermore, the selection of suitable additives is being explored to extend the shelf life of this bioproduct.

The ecological implications of using *B. cabrialesii* as a bioinoculant in commercial fields are also important questions that need to be answered. Metagenomic and ecological analyses of the modulation of the edaphic microbiome in fields where *B. cabrialesii* is employed serve as tools to understand these complex interactions within the microbiome.

Without a doubt, the promising uses of this novel bacterial species in sustainable agriculture are of great importance, and current and future research will help to fully harness its capabilities and bring it to the next level, formulating and commercializing a bioinoculant that could be used at the regional, national, or global level. Thus, with every discovery, more questions arise, and conducting new research and using diverse approaches to answer them are the main objectives of our research group. We conduct this work with the ultimate goal of contributing to the achievement of sustainable agriculture and global food safety.

## Figures and Tables

**Table 1:** Genome mining analysis of *Bacillus cabrialesii* TE3^T^. Biosynthetic gene clusters (BGC) regions identified by antiSMASH in the genome of strain TE3^T^ [13].

Region	From	To	Bgc Type	Most Similar Known Cluster	Similarity
Region 1	243,786	284,655	-	-	-
Region 2	332,686	354,098	-	-	-
Region 3	427,815	506,704	NRPS	Fengycin	100%
Region 4	590,916	696,061	NRPS	Bacillaene	100%
Region 5	1,288,334	1,309,116	-	-	-
Region 6	2,028,618	2,092,669	NRPS	Surfactin	86%
Region 7	2,222,460	2,263,350	Other	Rhizocticin A	93%
Region 8	2,740,198	2,781,616	Other	Bacilysin	100%
Region 9	2,784,080	2,805,691	RiPP	Subtilosin A	100%
Region 10	3,346,359	3,395,577	NRPS	Bacillibactin	100%
Region 11	4,047,919	4,078,040	NRPS	Plipastatin	38%
